# Comparison of Wound Education in Medical Schools in the United States, United Kingdom, and Germany

**Published:** 2008-01-11

**Authors:** Nima P. Patel, Mark S. Granick, Nikolaos K. Kanakaris, Peter V. Giannoudis, Frank Werdin, Hans-Oliver Rennekampff

**Affiliations:** Department of Surgery, New Jersey Medical School, University of Medicine and Dentistry of New Jersey, Newark, NJ; Division of Plastic Surgery, Department of Surgery, New Jersey Medical School, University of Medicine and Dentistry of New Jersey, Newark, NJ; Academic Department of Trauma & Orthopaedic Surgery, School of Medicine, University of Leeds, United Kingdom;; Department of Plastic, Hand, Reconstructive and Burn Surgery, University of Hanover, Hanover, Germany

## Abstract

**Objective:** Millions of patients are treated annually in the United States, United Kingdom, and Germany with either acute or chronic wounds. The purpose of this study is to compare how the medical education systems in the United States, Germany, and United Kingdom have prepared their physician trainees to deal with clinical issues of wounds. **Methods:** A retrospective study was performed in the United States by obtaining medical school curriculum data from the American Association of Medical Colleges, 2005. In the United Kingdom, data were obtained from the individual medical schools listed in the Royal Society of Medicine. In Germany, data were collected from a questionnaire sent to all the medical schools. **Results:** The total hours of required wound education received in the United States was 9.2 hours in the 4 years of medical school. In the United Kingdom, the total time devoted to wound-related issues equaled 4.9 hours over 5 years. In Germany, a total of 9 hours of wound education was provided over 6 years. **Conclusions:** Chronic wounds represent a serious problem for patients in terms of quality of life, lost employment time, and loss of income. Our comparison of the required wound education among the medical schools of United States, United Kingdom, and Germany demonstrated that all 3 systems are deficient in preparing future physicians to treat wound problems. We recommend that medical schools throughout the world devote a portion of their core curriculum to educating student physicians in the understanding of wound pathophysiology and treatment.

Acute and chronic wounds account for a large portion of interactions between patients and medical establishments in the United States, United Kingdom, and Germany. Chronic wounds affect 2.8 million patients in the United States.^1^ With the increased aging population in Germany, about 4 million patients suffer from chronic wounds.^2^ In these 3 countries, the vast majority of chronic ulcers derive from pressure ulcers, venous stasis ulcers, and diabetic neuropathic ulcers. Other causes of chronic wounds include osteomyelitis, operative wound dehiscence, infections, vasculitis, nonhealing traumatic wounds, and a variety of rare etiologies. In the United States, surgical site infections are the most common infections among postoperative patients,^3^ accounting for approximately 25% of all nosocomial infections.^4^ The prevalence of surgical site infections in the United Kingdom is 9%.^5^ Wound infection and delayed healing increase healthcare cost and lead to increased mortality and morbidity.

Wounds have a tremendous impact on the healthcare economy. In 2005, the United States spent more than $2.3 billion for advance wound care products. This expense is expected to rise at an average annual growth rate of 12.3% to $4.6 billion in 2011.^6^ In 1999, the Central Public Health Laboratory estimated the cost of hospital-acquired wound infection in the United Kingdom to be £930 million per year.^7^ The cost of wound care for German health insurances is 5 billion euros annually.^2^ In 1999, the cost of prescribing wound care products in the general practice in the United Kingdom exceederd £95 million.^8^ Comparably in the United States, basic wound healing products account for $1.1 billion.^6^

It is very surprising that clinicians are not well educated in wound care after taking into consideration the huge financial and clinical importance of the matter.^9^ In all 3 countries, little data exist to document the extent of wound education provided to medical students. This study employed the information provided by the American Association of Medical Colleges (AAMC) to determine the hours of directed wound education medical students receive in the United States. In the United Kingdom, data were collected from the individual medical school Web sites. In Germany, data were collected by surveys sent to medical school deans. The varied medical education systems as well as the repositories of curriculum data required the data acquisition process be different in each country.

## METHODS

### Data source

There was not a standard method of data collection among the 3 countries. In the United States, there is a central database, the AAMC. Such a database does not exist in either Germany or the United Kingdom.

In the United States, data were obtained from the AAMC database. The AAMC collects information regarding medical school curricula and accounts for all the hours dedicated to didactics, laboratory training, and clinical work. More than 6000 curriculum topics were documented among 100 American and Canadian medical schools. Only 50 American medical schools in 2005 documented any educational time dedicated to wound-related issues.

In the United Kingdom, data were obtained by evaluating the curriculum of each medical school individually as posted on their respective Web site. The Royal Society of Medicine provides a link to the Web site of each medical school, but does not acquire curriculum information. There are a total of 30 medical schools.

In Germany, written questionnaires were sent to the university deans regarding the wound care curriculum. Thirty-two of 36 German university deans responded.

### Population

The US study sample consisted of all American medical schools reporting curriculum data to the AAMC. The database included the institution name, course name, academic year, the session name, as well as the topics covered. Each session reported in the database represents an hour of didactic training on the topic.

The UK sample consisted of all the medical schools listed by the Royal Society of Medicine. The individual websites were accessed and the curriculum was searched using the search queries: “physiology of tissue injury,” “physiology of wound healing,” “wound management,” “clinical courses, wound healing,” and “clinical skills on wound management wound healing and stem cell biology.”

The Germany sample consisted of all the German universities that responded to the surveys. This study constituted analysis of publicly available data without identifiers and did not require institutional review board approval or informed consent.

### Medical school model

The medical school infrastructure is different in each of the included countries. In the United Kingdom, students generally commence their medical studies without any preliminary higher education. This contrasts with the US system, where a bachelor's degree is required for entry into medical school. The medical education itself takes 5 years, consisting of an aggregate of 2 years of preclinical training in an academic environment and 3 years of clinical training at a teaching hospital. The overall course of study is extended to 6 years if an intercalated degree is taken. After completion of clinical training, a student graduates as a bachelor of medicine and bachelor of surgery. In Germany, there is a 6-year curriculum after high school. The first 2 years are basic science years. Then, there are 3 years of lectures and seminars in nearly all clinical disciplines including emergency medicine, geriatrics, and others. The final year is spent at a hospital in 3 areas: surgery, internal medicine, and an elective. In the United States, medical school is 4 years after receiving an undergraduate degree, which can take 3 to 4 years. The first 2 years are generally basic science years and the last 2 years are spent at a teaching hospital.

### Data analysis

In the United States, 50 medical schools of the 100 American and Canadian schools listed by the AAMC were included in the study. Podiatric schools and osteopathic schools were excluded from the study. The 50 excluded schools had incomplete curriculum data in the AAMC. In the United Kingdom, there are a total of 30 medical schools. Of the 30 schools, data were available on 26 schools, regarding wound training in the first to third years, 24 schools had available data on the fourth year of training, and 25 schools had data on the fifth year of training. In Germany, of 36 universities, data were available on 32 (88%).

In the United States, elective courses that students took in their third and fourth years were not included in the study because they are not reported by the AAMC. Similarly, in the United Kingdom, 25% to 33% of the curriculum is allocated for student-selected courses. Elective courses in United Kingdom were not assessed because of the fact that their subjects may vary significantly between students even of the same program and of the same year of the studies. Likewise, in Germany, the sixth year consists of elective time. Consequently, these are not included in the study for all 3 countries. Also the bachelor of science courses in the sixth year of study are not included because they are not required to get the degree of bachelor of medicine and bachelor of surgery.

Wound education topics including physiology of wound healing, physiology of tissue injury, and clinical wound management were recorded as part of the total hours of wound education in the data analysis. All didactic courses, laboratories, and clinical experiences related to wounds were included in the study. The mean hours of wound education taught were divided according to each year of medical school training. In Germany, the hours of education were not divided according to year. The Germany data were only available for the total hours of education during the 6 years of training. The mean hours of training were compared among medical students in the United States, United Kingdom, and Germany within the limits imposed by the available data.

## RESULTS

**Figure 1:**
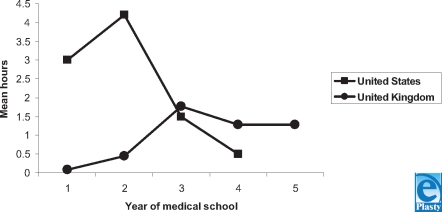
Mean hours of directed wound education. This graph shows the mean hours of directed wound education received by medical students in the United States and the United Kingdom. Note that the fifth year of training pertains only to the UK medical schools.

Figure 1 compares the total hours of directed wound education received by medical students in the United States and United Kingdom. The mean hours of total wound education in the United States during each year of medical school are 3.0, 4.2, 1.5, and 0.5 hours, respectively. A total of 9.2 hours of dedicated wound education is taught in American medical schools over 4 years.

In the UK medical system, a mean of 0.08 hours is taught in the first year and 0.46 hours in the second year. It increases to 1.77 hours in the third year and decreases to 1.29 hours in the fourth year. In the fifth and final year, 1.28 hours of wound education is taught. A total of 4.9 hours of wound training is provided to medical students in the United Kingdom over a 5-year period.

In Germany, a mean of 9 hours of wound education was provided to the medical students over the 6-year period. The mean hours of wound education per year was not available.

In the American schools, students receive more wound education in the first 2 years in comparison with students in the United Kingdom. In the third and fourth years, the UK students have a greater amount of wound education than the US students. In the American system, the greatest amount of wound education is received in the second year, 4.2 hours. In the British system, the greatest amount of wound education is received in the third year, 1.77 hours.

**Figure 2:**
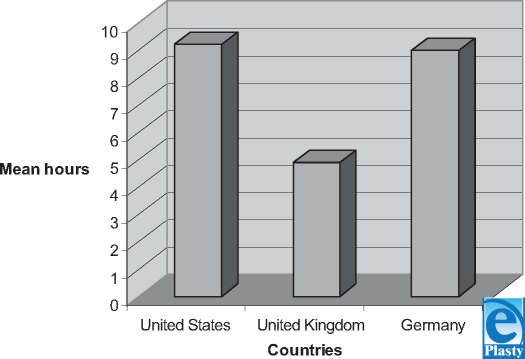
Mean hours of directed wound education in medical schools. This chart represents the total hours of wound education in each country. Note that in United States, it is over 4 years, in the United Kingdom, over 5 years, and in Germany, over 6 years.

Figure 2 shows that the American student receives more wound training (9.2 h/4 y) in comparison to their British (4.9 h/5 y) and German (9 h/6 y) counterparts. However, in all 3 systems, the elective courses that students decide to take are not included in the data analysis because these are not recorded by the AAMC. In the United Kingdom, the student-selected courses can be traced from the Web site but they do not represent the average student intake since they vary significantly. Therefore, it must be kept in mind that these hours are the minimum hours of education received in wound care. Those students who decide to take a dermatology or plastic surgery elective, for example, receive more wound education than the average student.

## DISCUSSION

Chronic wounds represent a major health burden and drain on healthcare resources.^10^ Studies have calculated the costs of wounds to the National Health Service to equal about £1 billion a year.^11^ In the United Kingdom, around 24,000 admissions a year are for diabetic foot ulcers, costing £17 million per year.^12^ Venous leg ulceration costs about £400 million annually in the United Kingdom.^13^

Similarly, in the United States, the cost of chronic wounds and the management of these wounds are enormous. The cost of diabetic ulceration is estimated at $150 million per year.^14^ The national cost of pressure ulcer treatment in the United States exceeds $1.335 billion per year.^15^ The sums reported both in these countries reflect only the direct costs and do not include the economic loss and the impaired quality of life experienced by patients with chronic wounds and their families.

We decided to measure the total number of hours of wound education medical students receive in the United States, United Kingdom, and Germany during their medical school training. The data show that very little education is provided to medical students on the topic of wounds, not only in the United States but also in the United Kingdom and Germany. In the United States, 78% of the total wound education received in medical school is provided in the first 2 years. On the contrary, in the United Kingdom, 89% of the total wound education is provided in the final 3 years of schooling. In all 3 systems, the total hours of wound education is marginal considering the huge clinical and economic burden that wounds have on society. In the United States, the least amount of directed wound education is provided in the fourth year that is the last year before residency training. In the United Kingdom, the least amount of wound training is provided in the first year.

While it is possible that students acquire additional wound training during the clinical surgery core rotation, this is not necessarily the case. In the United States, the educational division of the American College of Surgeons provides a curriculum for the core rotation. This involves 1 week of intensive didactic training that does not include wound treatment as one of the lectures. As far as clinical electives, additional exposure to wound care is not guaranteed.

Greater interest in wound management is needed among physician trainees to ensure higher standards of basic care. Wound-related education leads to improved communication, continuity of care, shortened hospital stays, and reduced costs.^16^ Lack of knowledge regarding wound care results in poor outcomes that can be reduced with increased education.^17^

Despite the tremendous impact of wounds on healthcare, few or no studies exist, documenting the amount of education medical students receive in wound management. There must be changes made in the medical student curricula to increase wound education at all levels. This study demonstrates that the lack of wound training is prevalent across two continents. We propose that more time is spent teaching wound management in medical schools. This could be achieved through workshops, lectures, home health nursing rotations, and clerkships as well as Web-based interactive programs, DVDs, and live or telecast symposia. By teaching students wound education in their preclinical years, they can apply this knowledge in their clinical years and care for the millions of patients that suffer from wounds.
